# Circularity in Polyamide Textiles: Enhancing Recycled Polymer Molar Mass with Carbodiimide Linear Coupling

**DOI:** 10.1021/acsomega.5c13446

**Published:** 2026-03-12

**Authors:** Graziela S. Baccarin, Mateus O. Costa, Rodrigo H. dos S. Garcia, Bruno Trebbi, Eduardo R. de Azevedo, Marco A. B. Ferreira, Lucas H. Staffa, Sandra A. Cruz

**Affiliations:** † Department of Chemistry (DQ), 67828 Federal University of São Carlos (UFSCar), Rodovia Washington Luis, km 235, São Carlos, São Paulo 13565-905, Brazil; ‡ Department of Materials Engineering (DEMA), Federal University of São Carlos (UFSCar), Rodovia Washington Luis, km 235, São Carlos, São Paulo 13565-905, Brazil; § Institute of Physics of São Carlos (IFSC), University of São Paulo (USP), Avenida Trabalhador São Carlense, 400, São Carlos, São Paulo 13566-590, Brazil

## Abstract

Textile waste from clothing production poses growing environmental and economic challenges with global fabric disposal expected to reach 148 million tons by 2030. Polyamide 6 (PA6), commonly used in fabrics, degrades during recycling due to hydrolysis, lowering its molar mass and limiting its reuse. This study presents a method to recycle PA6 fabrics using a carbodiimide (CDI) additive, which acts as an antihydrolysis agent and chain extender, aiming for cradle-to-cradle recycling. Controlling physicochemical properties, especially molar mass, is crucial; yet, the mechanisms of molar mass recovery are not well-understood and can be affected by humidity. This work showed that CDI reduced degradation in both dry and wet PA6, increasing molar mass by 40% and 75%, respectively, as confirmed by rheological analysis. Molecular investigation through ^13^C NMR showed no tertiary carbon signals, and Time Domain NMR indicated a higher glass transition temperature with CDI. These findings, supported by Density Functional Theory (DFT) calculations, suggest that CDI promotes linear chain extension over branching. This approach supports closed-loop recycling of PA6 fabrics, enhancing textile circularity and minimizing environmental impact.

## Graphical abstract

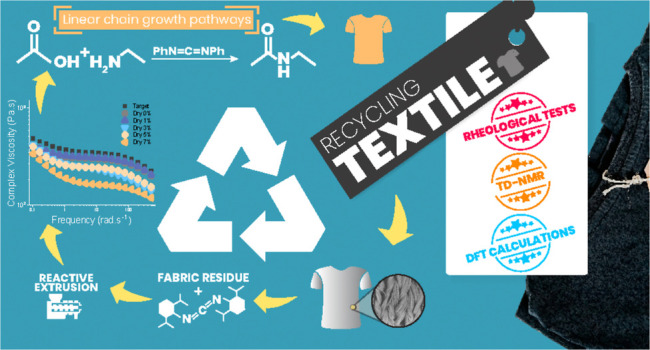

## 1 Introduction

The rise in living standards, global consumption, and economic growth and the expansion of the fast fashion industry have significantly impacted textile and clothing production, which has increased more than 6-fold over the past four decades, from 23.94 million tons in 1975 to 150.6 million tons in 2018.^1–5^ Furthermore, according to the 2023 World Trade Statistical Review,^6^ the textile and clothes export market accounted for approximately 6% of global manufactured goods exports in 2022, totaling USD 906 billion. China and Europe were the leading exporters, while the United States and Europe stood out as the main importers. This increase in the production is accompanied by an increase in the disposal, with 16.9, 16, and 20 million tons of textile waste being discarded in the USA, Europe, and China, respectively, in 2017.^7^

Of the total textile production, approximately 64% is derived from petrochemical-based materials. Once discarded, these textiles contribute to over 58 million tons of plastic waste annually, positioning the textile industry as the third-largest generator of plastic solid waste, surpassed only by the packaging and construction sectors.^1,2,7^ Of all textile waste generated, approximately 75% ends up in landfills or is incinerated.^3,4,8^ And less than 1% of the recycled material is reintegrated into textile production through a process known as closed-loop or cradle-to-cradle, highlighting the limited circularity currently achieved in the sector.^1,3,8–10^ Returning this material to its original application is a strategy aimed at reducing the input of virgin materials into the production process. This approach decreases the consumption of both renewable and nonrenewable resources, minimizes the use of hazardous substances, enhances process efficiency, and enables the production of high-value-added materials^9,11^ and is directly linked to the circular economy.

Polyamide (PA) is one of the most used materials in fabric production. According to the 2024 Textile Exchange Report,^12^ PA ranked as the third most utilized material in fiber production overall and the second among synthetic fibers in 2023. Among the various types of PA, polyamide 6 (PA6) was identified as the most employed for fiber manufacturing. Polyamides are characterized by the presence of amide groups in their structure, which renders them susceptible to hydrolysis reactions when exposed to moisture, particularly at elevated temperatures. These hydrolysis reactions result in polymer chain scission, reducing molar mass, and altering the molar mass distribution. Consequently, changes in material properties may occur, potentially hindering reprocessing and limiting the feasibility of returning the material to its original application or to high-value-added uses.^13–15^ The recycling of PA fabrics has been extensively examined in the literature, and most of which focus on (1) the separation and recovery of PA in mixed fabrics, with no further processing to improve properties,^16–18^ (2) the processing of mixed fabrics and the formation of polymer blends,^10,19,20^ and (3) the incorporation of other compounds into PA waste such as clay and graphene oxide, focusing the study on the effects of these compounds on the polymer matrix.^21,22^ However, there is a gap in research regarding the mechanical recycling of PA-based textile waste with a cradle-to-cradle approach, especially focusing on the adequacy of the molar mass and its distribution. The first challenge, however, lies in the reduction of the molar mass and the significant alteration in the rheological behavior of PA resulting from degradation processes. In this context, a class of compounds known as carbodiimides (CDIs) has emerged as a promising alternative, as they can react with terminal groups in PA chains, promoting chain extension and, consequently, increasing the molar mass. This effectiveness is attributed to their high reactivity with terminal amine and carboxylic acid groups, which are typically formed during degradation.^23,24^ Additionally, CDIs are derived from sterically hindered aromatic diisocyanates capable of reacting with water and are commercially used as antihydrolysis agents and hydrolytic stabilizers. Typically, this class of compounds is employed in the production and reactive modification of PA.^25–28^

The use of carbodiimides (CDIs) for molar mass modulation remains a topic rarely explored in the scientific literature, with the exception of the study conducted by Freitas et al.,^23^ which employed this class of compounds as chain extenders in recycled polyethylene terephthalate (PET). In the context of polyamides, investigations involving CDIs are still considerably limited and underdeveloped, despite the recognized potential of these additives to restore molar mass and, consequently, enable the reuse of these polymers in recycling processes. In particular, few studies have addressed the role of water as a reactive species or influencing agent in this process, and the available proposals concerning the reaction mechanisms between CDIs and polyamides are scarce and, for the most part, not clearly elucidated, except the work of Baccarin et al.,^29^ which is the first study related to the present paper. Furthermore, this gap extends to the mechanical recycling of polyamides to return to their original application, with most significant advances still concentrated on chemical recycling strategies.^30–33^

Thus, aiming to explore a novel strategy for the mechanical recycling of PA6-based textile waste, this study proposes the use of CDI as both a chain extender and an antihydrolysis agent, to regenerate the physicochemical and mechanical properties of the material by increasing its molar mass aiming to reintroduce the recycled resin in its original application (textile industry), thereby addressing the main challenges associated with conventional textile recycling. Moreover, this study focuses on the investigation of the reaction mechanism between CDI and PA6 and it also seeks to investigate the role of water in this reaction.

## 2 Experimental Section

### 2.1 Materials

The postindustrial textile waste used in this work was a PA6-based fabric provided by DIKLATEX (Americana, SP). The chain extender and hydrolysis stabilizer were monomeric CDI (Stabaxol I) provided by Lanxess Polymer Additives. The comparative PA6 (target) used was a virgin resin produced by BASF (Ultramid C 200).

### 2.2 Methods

#### 2.2.1 Recycling Process

In the initial stage, modifying the fabric’s form was necessary to facilitate the recycling process. The fabric was dried at 80 °C for 6 h in a vacuum oven (MMM, VACUCEL). Next, it was pressed at 240 °C, 15 tons for 5 min in a hydraulic press (LPB, Luxor) to form a rigid plastic sheet, which was then ground in a knife mill (Micro Powder System Cl, Batam) to obtain a granulated material. The granulated material was subsequently subjected to two distinct recycling processes, here termed (1) dry and (2) wet.

In the dry route, the granulated material was dried at 80 °C for 6 h in a vacuum oven (MMM, VACUCEL), and CDI was incorporated in varying proportions, as shown in Table 1. It was then processed in a corotating intermeshing twin-screw extruder (Process 11, Thermo Scientific, *D* = 11 mm; *L*/*D* = 40), with the screw profile presented in Figure 1, at 60 rpm, following this temperature set, in order, for each independent heating zone: 240 °C/240 °C/250 °C/250 °C/260 °C/250 °C/240 °C/240 °C.

**Table 1:** Formulations Used in the PA6 Fabric Recycling Process and the Samples Nomenclature^a^ ^,^ ^b^

route	PA6/fabric (wt %)	CDI (wt %)	material	sample
–	100^a^	0	Ultramid C 200	target
wet	100^a^	0	Ultramid C 200 recycled	extruded target
dry	100^b^	0	recycled pellet	dry 0%
	99^b^	1	recycled pellet	dry 1%
	97^b^	3	recycled pellet	dry 3%
	95^b^	5	recycled pellet	dry 5%
	93^b^	7	recycled pellet	dry 7%
wet	100^b^	0	recycled pellet	wet 0%
	99^b^	1	recycled pellet	wet 1%
	97^b^	3	recycled pellet	wet 3%
	95^b^	5	recycled pellet	wet 5%
	93^b^	7	recycled pellet	wet 7%

a PA6.

b Fabric.

**Figure 1:**
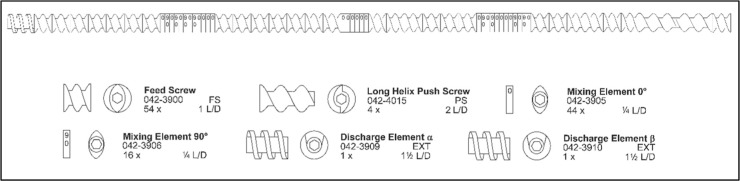
Screw profile utilized.

Finally, the polymer was pelletized (Varicut Pelletizer 11 MM, Thermo Fisher). The granulated material was incorporated with CDI without drying in the wet route, and the remaining processing steps were the same as those in the dry route. The diagram in Figure 2 summarizes the recycling process and illustrates the appearance of the fabric at different stages. The formulations and nomenclatures used are listed in Table 1.

**Figure 2:**
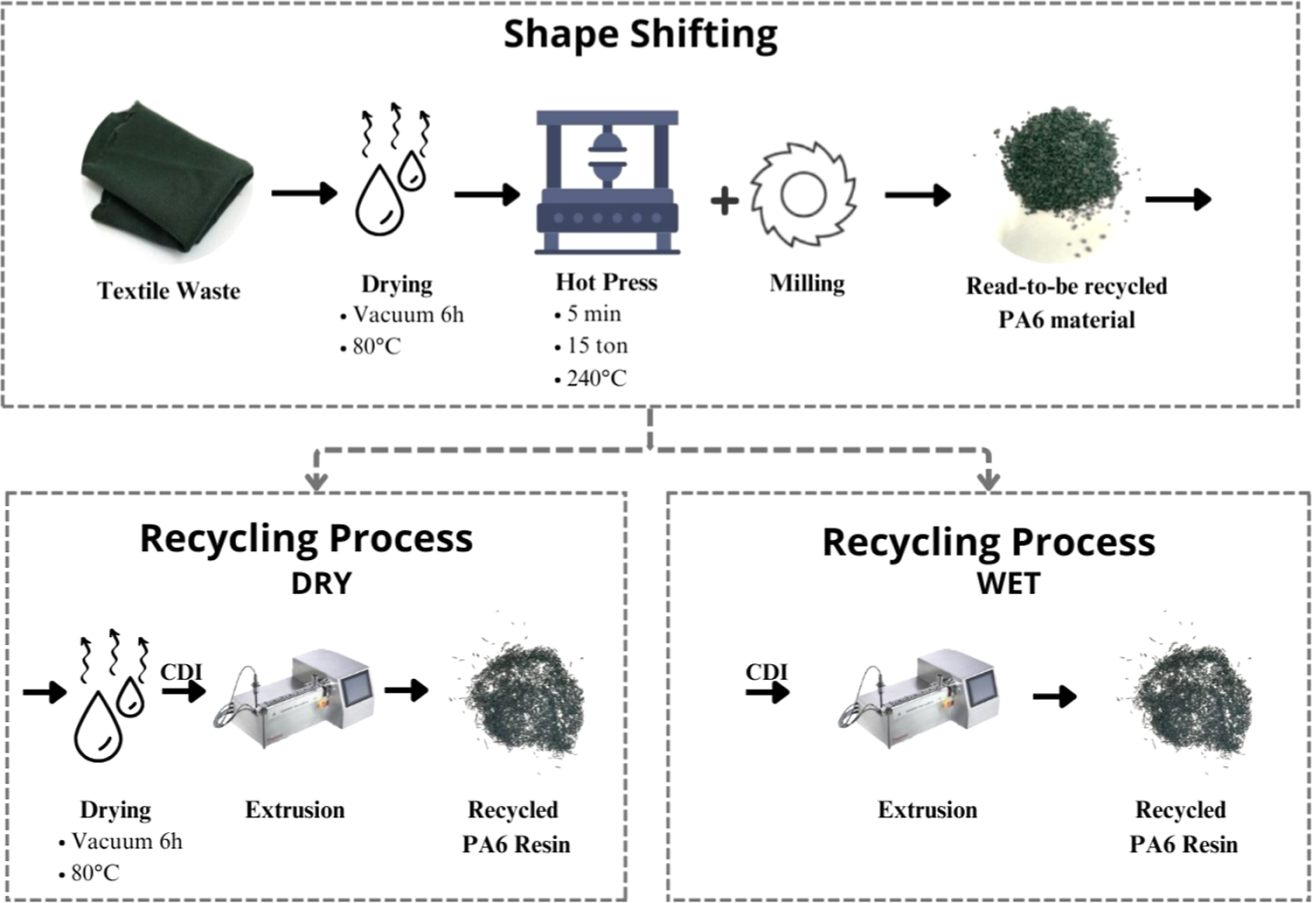
Diagram of the recycling process.

#### 2.2.2 Qualitative Analysis of the Initial Materials

To better characterize the initial materials, micrograph analyses and Fourier Transform Infrared Spectroscopy with Attenuated Total Reflectance (FTIR-ATR) analyses were performed. Micrographs were obtained using a FEG-SEM (XL30, Philips) to assess the morphology of the residue used as the starting material. The images were captured at a magnification of 60×, with an accelerating voltage of 3 kV. Prior to imaging, the samples were coated with a thin layer of gold to improve conductivity and image quality. FTIR-ATR (Nicolet 6700, Thermo Scientific) analyses were carried out to identify the chemical compositions of the initial materials. The samples were analyzed in film format, with each spectrum acquired by averaging 64 scans at a resolution of 4 cm^–1^, covering the wavenumber range from 400 cm^–1^ to 4000 cm^–1^.

#### 2.2.3 CDI Effect on Crystalline Phase and Crystallization Kinetics

To investigate the impact of carbodiimide (CDI) incorporation and processing on the crystalline structure and crystallization behavior of polyamide 6 (PA6), Differential Scanning Calorimetry (DSC), X-ray diffraction (XRD), and flow-induced crystallization analyses using parallel plate rheology were performed. DSC and XRD were used to examine changes in the crystalline phases and structure, and rheological analysis was conducted to study the crystallization kinetics under flow conditions. DSC analyses were performed using a power compensation calorimeter (200 F3, NETZSCH) in duplicate and under an inert atmosphere (N_2_ atmosphere). Prior to testing, samples were dried at 80 °C for 6 h under vacuum in a vacuum oven (VACUCEL, MMM) to eliminate moisture. During the DSC experiments, samples were heated from 20 to 300 °C at a rate of 10 °C/min, cooled to 20 °C at a rate of 20 °C/min, and subsequently reheated to 300 °C at a rate of 10 °C/min. XRD analyses were carried out using a D2 Phaser diffractometer (Bruker, model D2-204967) equipped with a normal focus Cu Kα radiation source, operating at a maximum output of 2 kW and featuring a vertical goniometer with a 185 mM scanning range. Diffraction patterns were collected in continuous scanning mode over a 2θ range from 4° to 120° at a scanning rate of 1°/min. The flow-induced crystallization tests were performed using a parallel plate rheometer (MC 302, Anton Paar) with 25 mM diameter plates, with a 1 mM plate gap, and under an inert nitrogen atmosphere, under steady-state conditions, with a shear rate of 3 s^–1^ and a temperature range from 240 to 185 °C.

#### 2.2.4 CDI Effect on Molar Mass and Its Distribution

To investigate the effect of CDI on molar mass and its distribution of the PA6, rheological tests were conducted using the same rheometer described earlier and under an inert nitrogen atmosphere. Before the tests, the samples were predried at 80 °C for 6 h in a vacuum oven (MMM, VACUCEL).

Flow sweep analyses were performed at 240 °C with a shear rate ranging from 0.01 s^–1^ to 100 s^–1^. Time sweep tests were conducted at 240 °C and 1 Hz with a 3% strain for 60 min. Frequency sweep analyses were carried out at 240 °C with a 3% strain and a frequency range of 0.01–500 rad/s. The 3% strain amplitude was previously confirmed to be within the linear viscoelasticity regime through a dynamic strain sweep at 240 °C and 1 rad/s, from 0.01% to 100%, as shown in the Supporting Information (Figures S1A–F and S2A–F).

#### 2.2.5 PA6 Chain Extension Mechanism Using CDI

To investigate the reaction mechanism between PA6 and CDI, time-domain nuclear magnetic resonance (TD-NMR), solution-state Nuclear Magnetic Resonance (NMR) spectroscopy, and Density Functional Theory (DFT) calculations were employed. TD-NMR analyses were conducted to monitor changes in the glass transition temperature (*T*_g_) induced by CDI incorporation and processing. ^1^H TD-NMR experiments were performed using a Mq20 Bruker MINISPEC spectrometer operating at 20 MHz, equipped with a 10 mM variable temperature probe. π/2 and π pulses were set at 2.4 and 4.7 μs, respectively, with a recycle delay of 1.5 s. A mixed Magic Sandwich Echo (MSE) pulse sequence, with an echo time of 100 μs, was applied prior to signal acquisition to minimize signal loss during the system dead time (13 μs). Dipolar Filtered Magic Sandwich Echo (DF-MSE) experiments^34^ were performed using filter times of 50, 100, and 200 μs. Sample temperature was controlled with a Bruker BVT 3000 system with a precision of ±1 K. Specific temperatures used in each experiment are detailed throughout the text.

Solution-state NMR analyses were conducted to investigate the potential formation of tertiary carbons during the recycling process. Samples were prepared by dissolving approximately 10 mg of PA6 in 0.5 mL of trifluoroacetic acid (TFA) and 0.1 mL of deuterated chloroform (CDCl_3_) for shimming purposes. Spectra were acquired using a Bruker AVANCE III spectrometer (100 MHz for ^13^C) with a 5 mM NMR tube. ^13^C NMR spectra were recorded with a 10.0 μs pulse width, a spectral window of 258.8267 ppm (26,041.75 Hz), 33,768 scans, a relaxation delay of 0.10 s, and an acquisition time of 0.6291 s, at a temperature of 26.0 °C, with 16,384 data points and a resolution of 0.40 Hz.

DFT calculations were performed to determine the most energetically favorable reaction pathways, whether they favor linear chain extension or branched structure formation. Initial molecular structures were generated from SMILES strings using OpenBabel (version 3.1.1)^35^ and preoptimized via the semiempirical extended tight-binding method implemented in xTB (version 6.7.0).^36^ Subsequent geometry optimizations and frequency analyses were carried out using Gaussian 16, Revision C.01., at the M06-2X/def2-TZVP level of theory.^37,38^ The nature of each stationary point was verified by vibrational analysis, ensuring zero imaginary frequencies for minima and one imaginary frequency for the transition states. Thermal corrections to Gibbs free energies were derived from vibrational analyses at 298.15 K and 1 atm. Solvent effects were modeled using the SMD implicit solvation model, with a custom dielectric constant (ε = 3.2) to simulate the dielectric environment of molten polyamide.^35^ Final free energy values are reported in kcal·mol^–1^.

#### 2.2.6 CDI Effect on the Mechanical Properties

To understand the influence of CDI and humidity on the mechanical properties of PA6, tensile tests were carried out. For specimen preparation, the materials were dried at 80 °C for 6 h under vacuum and pressed at 240 °C. The samples were prepared by stamping based on the ASTM D882-2012 standard for thin films. Tensile tests were performed in quintuplicate using a universal testing machine (5569, Instron), with a speed of 10 mM·min^–1^, with a 500 N load cell, and at room temperature. From this test, the strain stress, yield stress, and Young’s modulus were determined.

## 3 Results and Discussion

### 3.1 Qualitative Analysis of the Initial Materials

The first challenge related to textile recycling is identification of the initial materials. Most of the time, the label is not faithful to all of the materials used to produce the final product. So, a precise analysis was made, aiming to identify and verify the materials used in the production of the textile waste and the CDI that was used in this work. Regarding pigmentation and additives used in textile production, these factors can influence reprocessing and recycling, often acting as potential catalysts for chain scission. Regarding pigmentation and additives used in textile production, these factors can influence reprocessing and recycling, often acting as potential catalysts for chain scission. However, in the present study, all comparisons were performed using samples derived from the same starting material; therefore, the effects of pigmentation and additives were not considered.

Figure 3 presents the SEM and DSC curves of the fabric residue images of the CDI used, NMR, and the chemical structure of CDI, as well as the FTIR images of CDI, the fabric residue, and the target.

**Figure 3:**
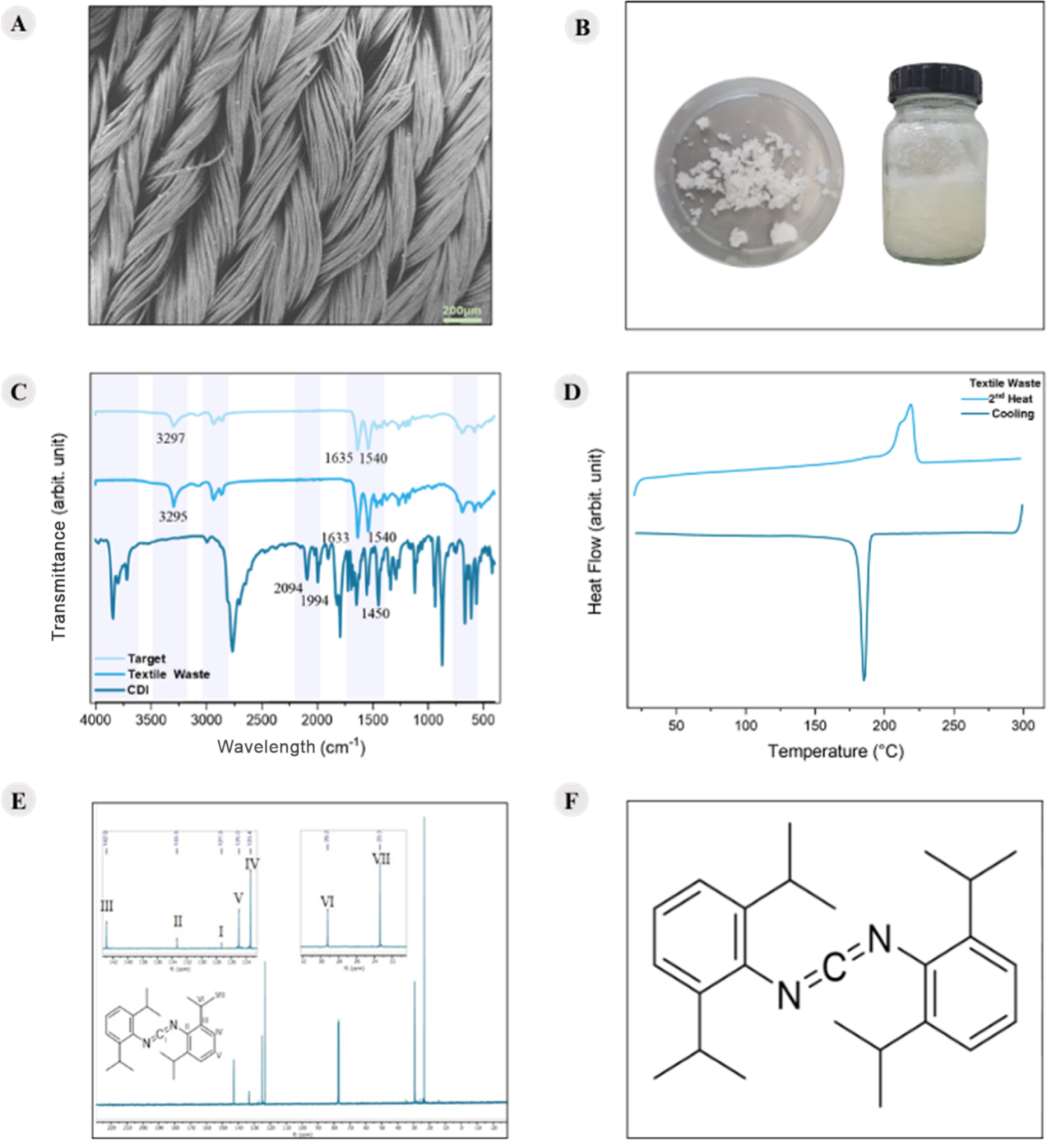
(A) SEM of fabric residue; (B) images of CDI; (C) FTIR spectra of the target, fabric residue, and CDI; (D) DSC curves of the fabric residue referring to the 2nd heating and cooling; (E) ^13^C NMR spectrum of CDI in CDCl_3_ (100 MHz)—The whole spectrum is presented in Figure S3 of the Supporting Information; (F) chemical structure of CDI.

Figure 3A shows that the textile residue consists of woven fibers, while Figure 3B shows the CDI used, which according to the manufacturer is a liquid crystal. Table 2 presents the functional groups corresponding to the main peaks of the FTIR-ATR spectrum shown in Figure 3C.

**Table 2:** Compounds, Functional Groups, and Their Respective Characteristic Wavenumbers of the FTIR Spectrum^a^

compound	functional group	wavelength (cm^–1^)
PA/CDI	C–H (bending)	600–1000
CDI	C=N (stretching)	1640–1690
CDI	C–C (stretching)	1450–1600
PA	N–H (bending)	1550–1640
PA	C=O	1630–1690
CDI	–N=C=C–N–	1994/2094
PA/CDI	C–H (symmetric/asymmetric stretching)	2853–2962
CDI	Ar–H (stretching)	3030
PA	N–H (stretching)	3300–3500

a FTIR band assignments of the analyzed compounds based on literature data refs 15, 39, and 40 .

The analysis of Figure 3C and the assignments presented in Table 2 confirm that the textile residue is composed of polyamide (PA). This identification is supported by the presence of characteristic peaks in the FTIR spectrum, particularly at 3295/3297 cm^–1^, attributed to the N–H stretching vibration, at 1540 cm^–1^, attributed to the N–H stretching vibration of amide II, and at 1633/1635 cm^–1^, corresponding to the C=O stretching vibration, which are typical of the chemical structure of polyamides.^15,40^ However, this technique does not accurately identify which specific PA was used in the fabric’s production. To address this, DSC tests were conducted (Figure 3D). The DSC curves show a melting event at 219.84 °C and a crystallization event at 185.38 °C, which corresponds to the *T*_m_ and *T*_c_ of PA6, respectively.^17,41^ Thus, it can be concluded that the textile residue is a fabric based on PA6.

Additionally, based on the FTIR analysis (Figure 3C) and the assignments presented in Table 2, it is possible to identify the additive as an aromatic carbodiimide, as especially the peaks at 3030 cm^–1^ (Ar–H stretching), the double peaks at 1994 cm^–1^ and 2094 cm^–1^ (–N=C=C–N–), and the peaks at 1450 cm^–1^ (C–C stretching).^39^ However, this technique does not give further information about the CDI chemical structure. So, to understand its chemical structure, an NMR analysis was performed. The ^13^C NMR spectrum of commercial CDI (Figure 3E) displays a clean profile, with all observed signals fully assignable to the CDI structure, indicating its high purity and confirming the identity of the compound used.^36^ The assigned signals are as follows: sp-hybridized carbon a appears as a low-intensity peak at 127.3 ppm. The quaternary sp^2^ carbons b and c are observed at 133.3 and 142.9 ppm, respectively, with c shifted upfield due to the ortho effect. The more shielded aromatic carbons d and e are observed at 123.4 and 125.0 ppm, respectively, with e upfielded due to the para effect. The alkyl carbons f and g are observed at 29.2 and 23.3 ppm, respectively, with f being less shielded due to anisotropic effects.

### 3.2 CDI Effect on Crystallinity and on Crystallization Kinetics

Changes in material properties accompany the reprocessing process and are closely linked to alterations in the molar mass and its distribution. Among the properties that may be affected are the crystalline phase of the material and the kinetics of crystallization. The fiber production process inherently results in flow-induced crystallization, making its evaluation essential under nonquiescent conditions. Figure 4 presents the spectra obtained through XRD tests, the second heating curves from DSC, and the flow-induced crystallization curves.

**Figure 4:**
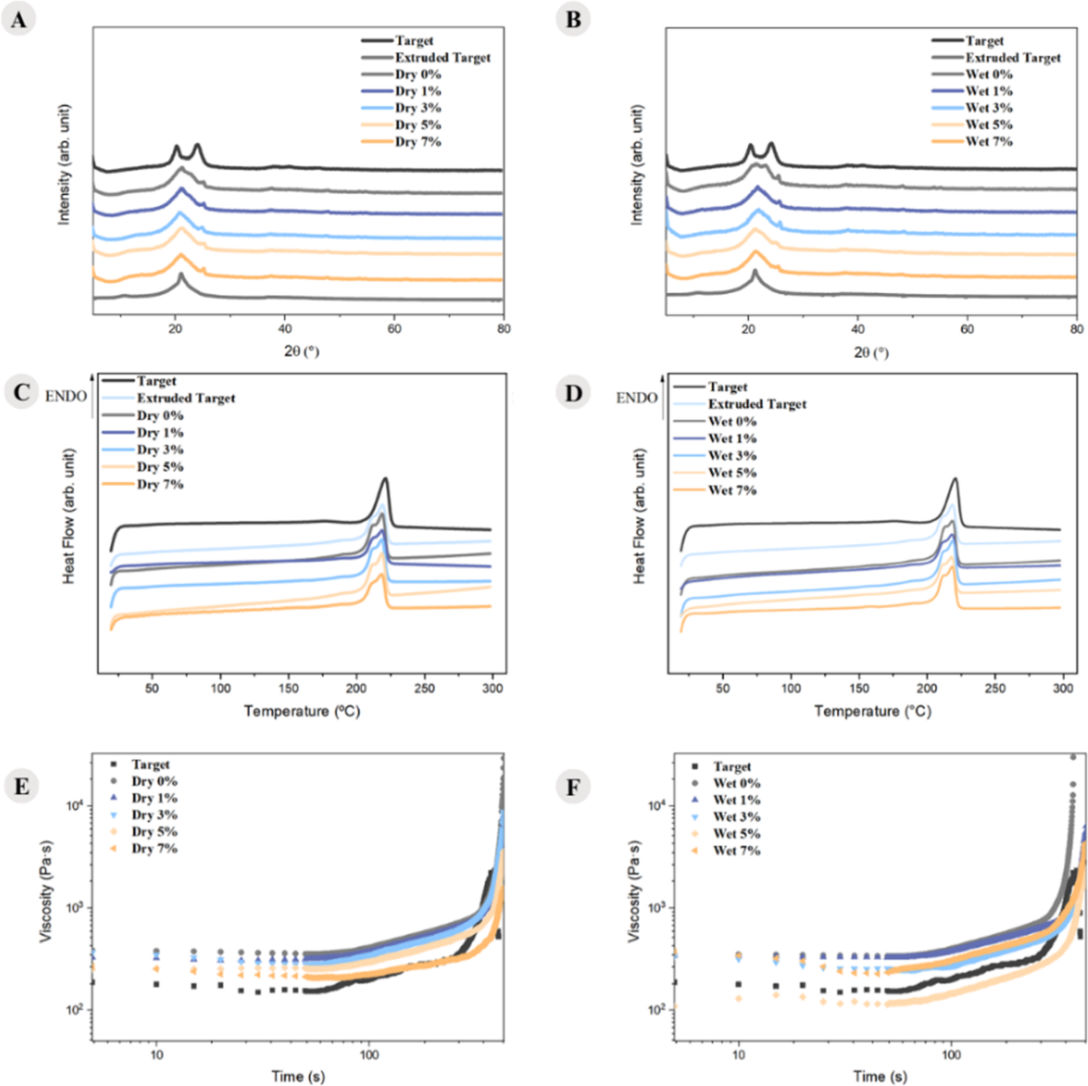
XRD spectra: (A) dry route and (B) wet route, curves corresponding to the 2nd heating of DSC: (C) dry route and (D) wet route, and flow-induced crystallization curves: (E) dry route and (F) wet route.

Aliphatic polyamides, such as PA6, have two distinct crystalline structures known as α and γ, which arise from the material’s significant ability to form hydrogen bonds in the crystalline phase. A considerable fraction of these bonds remains unchanged even in the molten state.^42,43^ The α configuration, the most stable of the two, has a fully extended structure, resulting in antiparallelly oriented chains. Consequently, the amides and methylene units are in the same plane, with hydrogen bonds between the chains, forming a pile of sheets connected by these bonds and resulting in a monoclinic crystal.^42^ The γ configuration, on the other hand, has a 60° twist relative to the α configuration, which results in the amides and methylene units being in different planes, leading to a crystal similar to a hexagonal structure, referred to as pseudohexagonal. Additionally, the crystalline structure that PA6 assumes when transitioning from the molten state to the solid state depends on various factors, such as thermal conditions, applied stress, moisture content, and additives.^42^

From Figure 4A,B, it is possible to observe that the target sample exhibits two distinct peaks, the first around 20° and the second around 24°, which characterize the α form. The extruded target sample and those with CDI, both wet and dry, display a peak around 22° and a shoulder around 25°, which characterizes the γ form.^43,44^ However, the samples with 0% CDI show a mixture of both forms, indicating partial conversion from the α form to the γ form. This suggests that CDI and the recycling process caused PA6 to change its crystalline phase. In Figure 4C,D, it can be observed that the samples that underwent the extrusion process show a shoulder in the peak corresponding to the melting of PA6 in both sample types, while the target sample does not, further indicating that the processing caused PA6 to change its crystalline phase. This is because the α configuration does not show the shoulder in the peak corresponding to the melting event in the DSC, whereas the γ configuration does.^42^

During the extrusion process, the material is subjected to shear flow, and consequently, the crystallization process is flow-induced. Rheometry allows one to obtain information about the material’s nonquiescent nucleation kinetics, as it is inversely proportional to the nucleation start time. In other words, the longer the nucleation start time, the slower the material nucleation kinetics,^45–48^ and shorter crystallization times indicate structural changes that facilitate nucleation and crystallization. Conversely, higher molar mass hinders crystallization, leading to longer crystallization times.^45,47,49,50^ Analyzing the curves obtained through the rheology tests of flow-induced crystallization (Figure 4 E,F), CDI increases the nucleation start time for both sample types. Therefore, the use of CDI hinders the nonquiescent crystallization kinetics of PA6, indicating that the addition of CDI decreases the polymer chains mobility.^46–48^

A similar behavior was observed in the work of Fitaroni et al.,^46^ who reported that contamination in PP samples, induced by a prepared cocktail (10% chloroform, 10% toluene, 1% tetracosane, and 1% benzophenone dissolved in 78% *n*-heptane), reduced the nucleation start time and consequently accelerated nucleation kinetics. They attributed this effect to contaminants decreasing chain entanglement, which increased the molecular mobility. In the present study, the same reasoning applies to chain mobility governing the nucleation process. However, instead of contamination reducing entanglement, the increase in molar mass of PA6 samples with CDI promoted greater chain entanglement, decreased chain mobility, and therefore increased the nucleation starting time. Thus, both works highlight the same underlying mechanism.

### 3.3 CDI Effect on Molar Mass and Its Distribution

As mentioned previously, the recycling of postindustrial textile waste was carried out through two distinct routes, referred to as dry and wet. Structural changes over time were analyzed through oscillatory rheology tests using a time sweep, and frequency sweep analyses were conducted to evaluate the effects of CDI and moisture on the molar mass during the PA6 fabric recycling process. Both analyses are presented in Figure 5.

**Figure 5:**
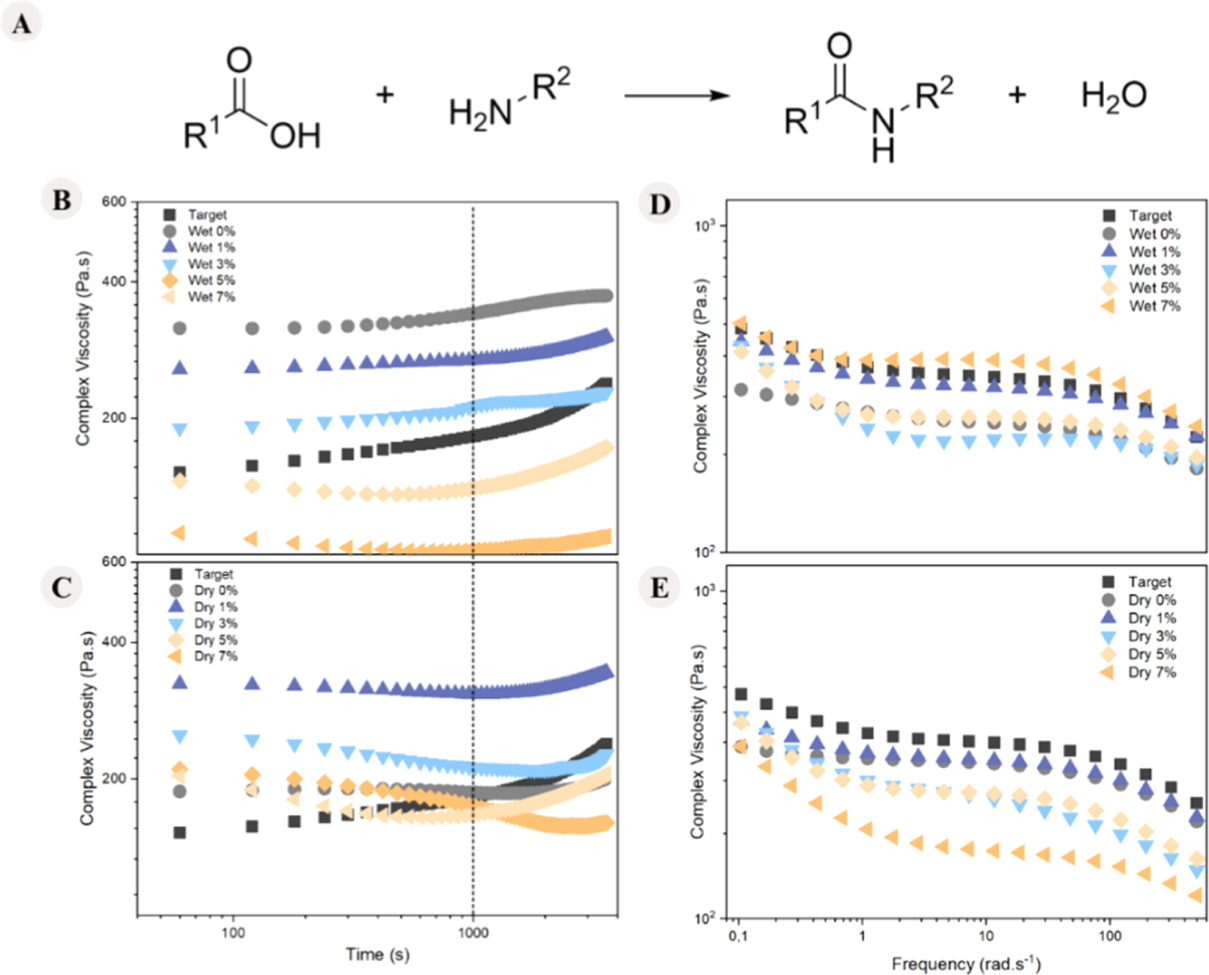
(A) Condensation reaction of a carboxylic acid and an amine; time sweep curves of (B) wet and (C) dry routes; frequency sweep curves of (D) wet and (E) dry routes.

Analyzing the results presented in Figure 5B,C, it is evident that at the beginning of the experiment, no significant changes in complex viscosity were observed for any of the samples, indicating that no reactions occurred during this initial phase. Additionally, for both types of samples, a noticeable change in slope is observed around 1000 s in almost all curves, suggesting that condensation reactions are likely to occur after this point. In these reactions, the carboxylic acid chain end reacts with the amine chain end, forming an amide bond and releasing a water molecule, as illustrated in Figure 5A, ultimately leading to an increase in the molar mass. Consequently, only the data obtained up to 1000 s were considered for the other sweep modes. A similar effect was observed in the oscillatory rheology time sweep study by Freitas et al.,^23^ where an increase in PET viscosity over time was noted, similarly to PA6, as both are condensation polymers. Also, in Figure 5D,E, it can be observed that at low frequencies, for both types of samples, there was an increase in complex viscosity with the addition of CDI, except for the dry 7%. The samples that most closely matched the complex viscosity of the target material were the wet 7% and dry 3%.

Table 3 presents the complex viscosities at lower frequencies obtained from oscillatory rheology frequency sweep tests along with the corresponding ratios, which were calculated by dividing the complex viscosity of each sample by that of the 0% CDI sample.

**Table 3:** Data on Complex Viscosities (η*) Obtained from Oscillatory Rheometry Frequency Sweep Tests for Dry and Wet Samples^a^

sample	dry	dry	wet	wet
	η* (Pa.s)	$\frac{\eta_{sample}^{*}}{\eta_{0\%}^{*}}$	η* (Pa.s)	$\frac{\eta_{sample}^{*}}{\eta_{0\%}^{*}}$
target	540.14	1.53	540.14	1.62
0% CDI	352.18	1.00	332.67	1.00
1% CDI	445.07	1.26	492.81	1.48
3% CDI	494.26	1.40	528.60	1.59
5% CDI	476.77	1.35	498.94	1.50
7% CDI	410.60	1.17	582.52	1.75

a $\frac{\eta_{sample}^{*}}{\eta_{0\%}^{*}}$ —complex viscosity of each sample by that of the 0% CDI sample.

The complex viscosity at higher frequencies is influenced by the broadening or narrowing of the molar mass distribution and/or the presence of chain branching.^41,51–54^ At lower frequencies (approaching zero), the viscosity is mainly related to the average molar mass.^51–53^ Based on this, Table 3 supports and reinforces the trends observed in Figure 5, making it more straightforward that for the dry samples, there is a tendency for the complex viscosity, and consequently the molar mass, to increase up to 3% CDI. Beyond 5% CDI, however, a decreasing trend is observed. The complex viscosity that most closely matched the target sample was 3% CDI. This result corresponds to a 40% increase in molar mass compared to the sample processed without CDI.

In the wet route, there is a trend of increasing complex viscosity with higher CDI content, with the 7% CDI sample showing a higher complex viscosity than the target sample, indicating that its molar mass is greater. The wet sample with 7% CDI showed a 75% increase in complex viscosity compared with the wet sample processed without CDI. These results indicate that CDI acted to prevent degradation and functioned as a chain extender in all samples. Based on these results, particularly those related to the molar mass ratio, there is evidence suggesting that CDI performs better in the presence of water. This could be a breakthrough, as using this method and additive could eliminate the drying steps for PA6, reducing energy consumption, processing time, and cost.

The same chain extension effect and better performance in wet samples were observed for PET in the study by Freitas et al.^23^ It was found that PET exhibited higher complex viscosities and, consequently, higher molar masses with increasing CDI content. Furthermore, it was observed that samples not subjected to drying prior to processing and therefore with higher moisture content showed higher molar masses, indicating a greater efficiency of CDI in environments with elevated water content. The present study suggests that the presence of water is a key factor for the better performance of CDI as a chain extender.

Flow sweep analyses were performed and are presented in Figure 6, accompanied by a schematic representation of the polymer chain behavior during the rheological testing.

**Figure 6:**
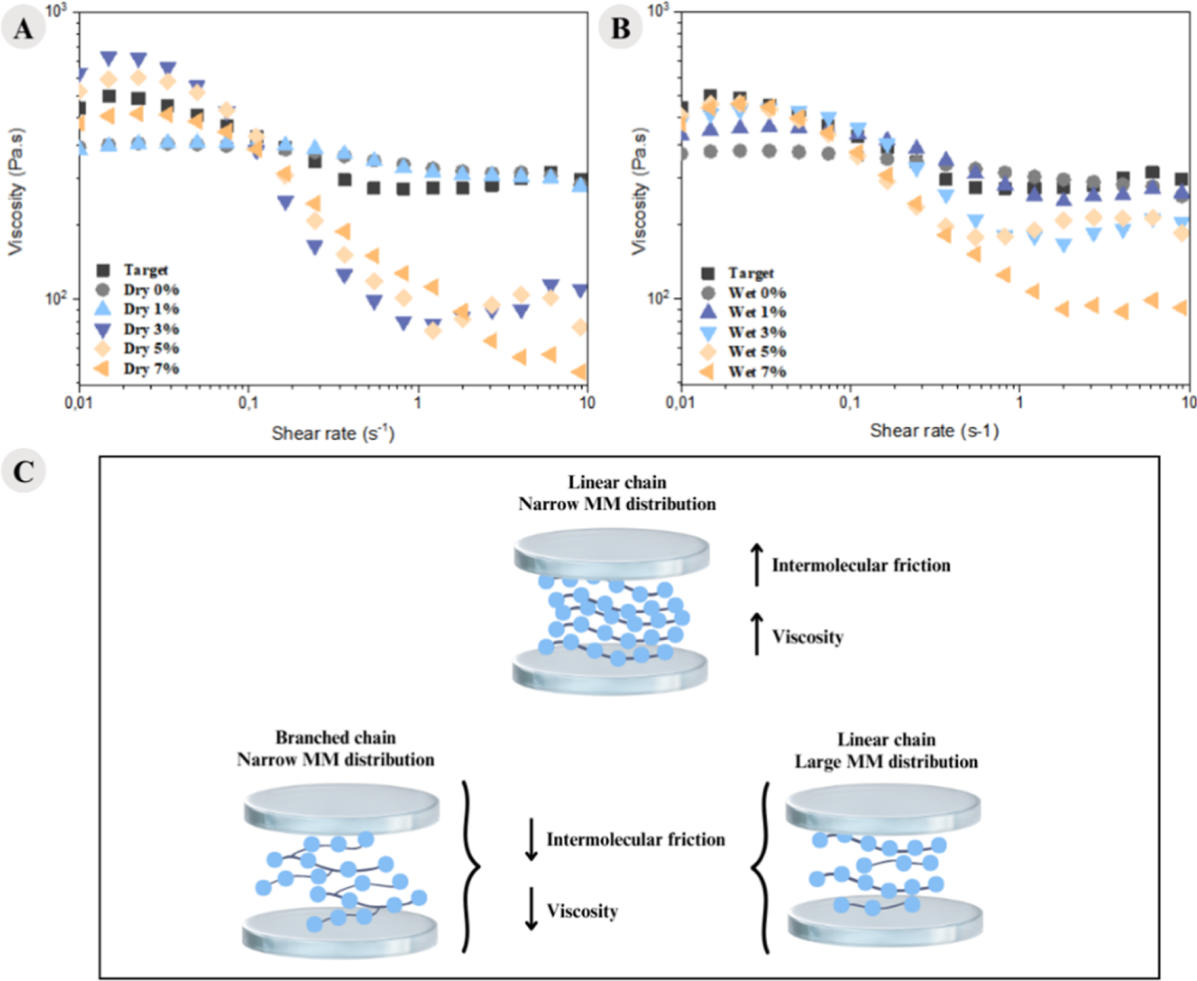
Viscosity as a function of shear rate for the samples obtained by the (A) dry and (B) wet routes, and (C) schematic diagram of polymer chain behavior during the rheology test.

Analyzing the rheological curves under steady-state conditions (Figure 6A,B), it is evident that shear-thinning behavior occurs, characterized by a decrease in viscosity at high shear rates for the samples containing 3%, 5%, and 7% CDI, in dry and wet conditions.

Furthermore, this behavior becomes more pronounced with increasing CDI concentration, suggesting an increase in the molar mass. However, this increase may be associated with the formation of a greater number of branches or with the broadening of the molar mass distribution.^24,41^ These two properties cannot be differentiated through rheological analysis because in rheological analyses, the viscosity of polymer samples depends on the friction between polymer chains; the higher the friction, the higher the viscosity. When the molar mass distribution broadens, shorter chains align with the flow and occupy the spaces between longer chains. The resulting free volume, particularly at the chain ends, separates the chains, reducing friction and thus viscosity. Similarly, an increase in branching also increases the free volume, which separates the chains, reduces friction, and lowers viscosity and consequently increases the shear thinning behavior,^24,41^ as shown in the schematic in Figure 6C. This behavior was more pronounced in the dry samples, which also indicates better CDI performance in environments with higher water content.

The intensification of shear thinning behavior with the addition of chain extenders in PA6 was also observed by Cai et al.^41^ The authors used a triepoxy small molecule as a chain extender for PA6 in different proportions. It was observed that chain extension, in addition to increasing the molar mass, enhanced the shear thinning behavior of the material, which was attributed to the presence of long branches.

Blanco-Díaz et al.^54^ simulated the rheological behavior of different molar mass distributions, fixing the average molar mass of PE. They observed a decrease in viscosity with an increase in shear rate, indicating an increase in shear thinning behavior with a broader molar mass distribution.

### 3.4 Chain Extension Mechanism of PA6 Using CDI

Time domain NMR is widely used to investigate the molecular and microstructural properties of various materials.^34,55–59^ By utilizing a pool of techniques, TD-NMR is particularly effective in detecting mobility changes due to its sensitivity to motional parameters, as evidenced by changes in relaxation times in liquids or alterations in the signal characteristics during solid–liquid (rigid-mobile) transitions.

The signal observed in TD-NMR strongly depends on the presence of motion within the tens of kHz frequency range. This is due to the orientation-dependent magnetic dipolar interactions between the ^1^H nuclei.^60,61^ The strength of these interactions is inversely related to the cube of the ^1^H–^1^H distances, reaching up to 50 kHz in typical polymer systems, such as PA. Moreover, these interactions depend on the molecular orientation relative to the applied magnetic field, resulting in a wide frequency dispersion in the NMR spectra, as the ^1^H nuclei in different molecular segments experience different dipolar interactions. This frequency dispersion leads to an NMR signal that decays within a few microseconds and exhibits a Gaussian-like shape.^56^ However, orientation dependence also makes the dipolar interaction highly sensitive to molecular motions with rates above 50 kHz, which can average out the interaction, effectively making it invisible in the NMR signal. In such cases, the signal typically decays with an exponential-like shape on the millisecond time scale. Thus, while rigid molecular segments give rise to time-domain NMR signals with fast decay and Gaussian-like shapes, mobile molecular segments produce signals with a slow exponential decay.

Mauss and Saalwächter explored the motion-induced contrast in the TD-NMR signal in ref 61, where they proposed a multicomponent fit function consisting of three components: a Gaussian-like decay to represent the rigid segments, a stretched exponential decay to represent the mobile segments, and a sub-Gaussian decay to represent segments with intermediate mobility. Indeed, for a semicrystalline polymer at a temperature above the glass transition but below the melting point, the rigid Gaussian decay corresponds to the crystalline region signal, the stretched exponential decay corresponds to the amorphous region signals, and the sub-Gaussian decay corresponds to the signal from segments in the crystalline–amorphous interphase. This was used to investigate the microstructure of polyethylene and many other polymer systems.^58,59,62,63^ However, a simplified version of this model, considering only the Gaussian and stretched exponential components, can also be applied in cases where the interest lies solely in evaluating the change in amorphous chain mobility.

Thus, in the current investigation, the NMR signal was acquired after the application of the mixed-MSE echo pulse sequence (see the rationale for its use in the experimental section). The measurements were conducted at 198 °C, above the glass transition temperature, and just below the melting point. Therefore, at this temperature, the segments in the crystalline phase remain rigid from an NMR perspective. The signal (normalized by its maximum intensity) was fitted using a double-component decay like^62^

1 $$\frac{s\left(t\right)}{s\left(0\right)}=f_{r}e^{-{\left(\frac{t}{T_{2r}}\right)}^{2}}+f_{m}e^{-{\left(\frac{t}{T_{2m}}\right)}^{\beta }}$$

where *f*_r_ *e* and *f*_m_ *e* are, respectively, the faction segments in the rigid (crystalline phase) and mobile (amorphous) domains. *T*_2r_ and *T*_2m_ define the decay time of the signals and β is the stretching parameters for the exponential decay.

Figure 7A,B shows the ^1^H TD-NMR signals acquired for samples target, dry 0%, dry 3%, wet 0%, and wet 7%. The parameters extracted from the fit are listed in Table 4. As can be noticed, the most significant trend is the decrease in the decay time of the signals from the amorphous component in the samples dry 3% and wet 7% compared with the 0% counterparts. This suggests that there is a slowdown of the motions in the amorphous domains of the samples induced by CDI, which motivates a more detailed study on the mobility of these segments.

**Figure 7:**
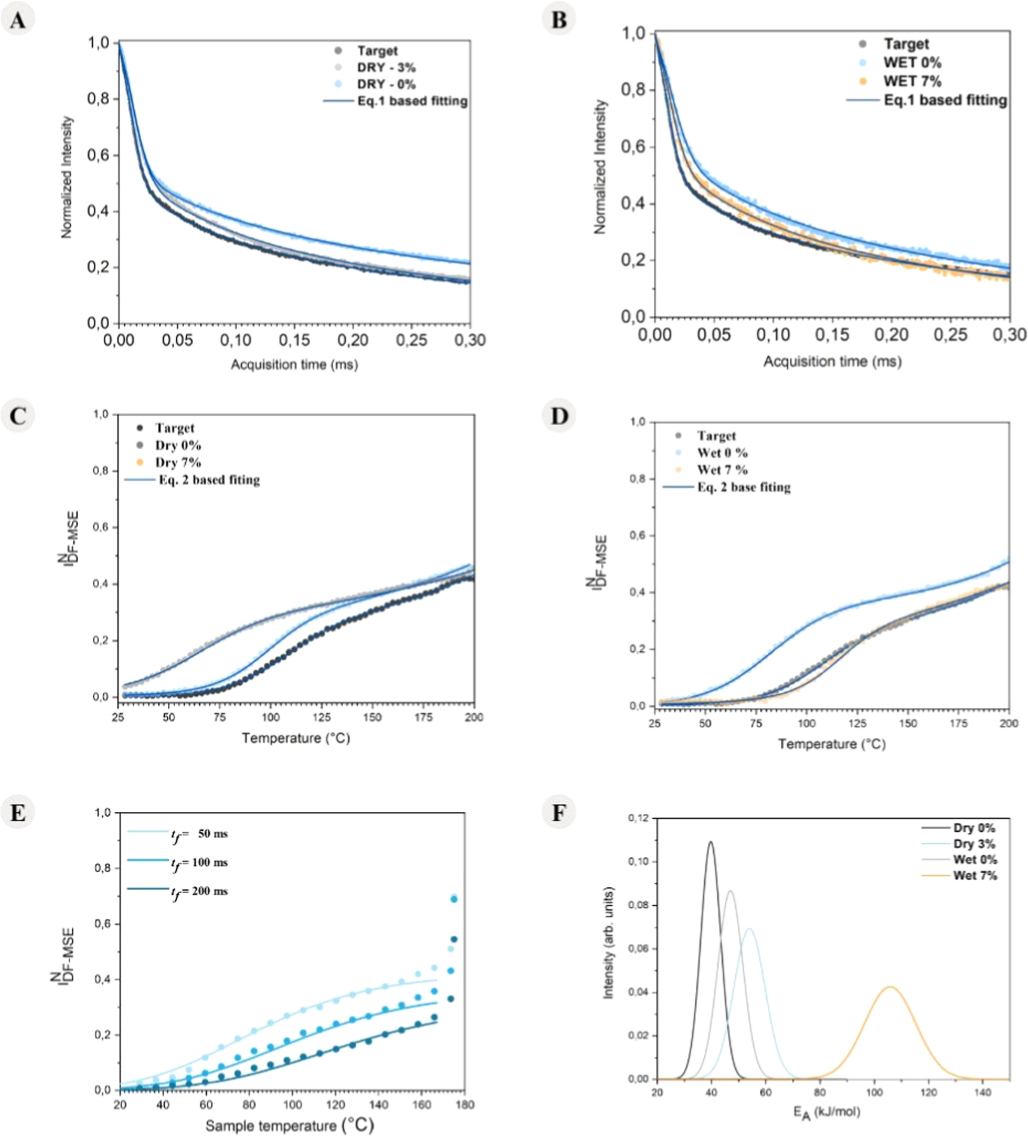
TD-NMR signals at 198 °C and fitting curves for the (A) dry route and (B) wet route. *I*_DF‑MSE_^N^ vs *T* curves and fittings curves for the (C) dry route and (D) wet route. (E) Plot of the normal distribution of apparent activation energies obtained for fitting *I*_DF‑MSE_^N^ vs *T* experimental curves. (F) The fitting parameters used to build the plots are shown in Table 5 and the full data set and fitting curves are provided in Supporting Information Figure S4.

**Table 4:** Parameters Resulting from Fitting the NMR Signals in Figure 7A Using Eq 1

sample	*f*_r_	*T*_2r_	*f*_m_	*T*_2m_	β
target	0.36 ± 0.01	0.0154 ± 0.0001	0.64 ± 0.01	0.150 ± 0.002	0.61 ± 0.01
dry 0%	0.34 ± 0.01	0.0173 ± 0.0001	0.66 ± 0.01	0.235 ± 0.003	0.65 ± 0.01
dry 3%	0.33 ± 0.01	0.0176 ± 0.0001	0.67 ± 0.01	0.158 ± 0.002	0.71 ± 0.01
wet 0%	0.29 ± 0.01	0.0206 ± 0.0001	0.71 ± 0.01	0.172 ± 0.003	0.74 ± 0.01
wet 7%	0.32 ± 0.01	0.0178 ± 0.0001	0.68 ± 0.01	0.152 ± 0.003	0.75 ± 0.01

To further investigate the changes in the mobility of the amorphous phase segments with CDI, the Dipolar-Filtered Magic Sandwich Echo (DF-MSE) NMR technique was used. Simply put, this technique relies on adding the so-called Goldman–Shen (GS) pulse sequence prior to the mixed-MSE echo sequence. The GS pulses act as a dipolar filter (hence the name DF-MSE), attenuating the signals from segments that experience strong ^1^H–^1^H dipolar interactions, i.e., rigid segments, depending on the adjustable duration of a pulse sequence parameter called the filter time *t*_f_.^34^ For filter times of >50 μs, the signal from rigid segments is almost completely suppressed, leaving only the signal from mobile segments. By normalizing the intensity of this filtered signal with the intensity of a second acquisition (where the filter time is set to zero, allowing detection of all segments), a normalized intensity *I*_DF‑MSE_^N^ can be obtained. This quantity is proportional to the fraction of mobile segments, *f*_m_.

The DF-MSE experiments can be done as a function of temperature to provide *I*_DF‑MSE_^N^ vs *T* curves, monitoring the change on *f*_m_ as the temperature changes. Thus, if there is a change in the mobility of the segments as a function of temperature, it can be directly detected. In other words, *I*_DF‑MSE_^N^ vs *T* curves probe the onset of molecular motions that average the ^1^H–^1^H dipolar interaction, i.e., with rates higher than dipolar interaction (∼50 kHz in typical organic polymers).

In the case of the PA samples analyzed here, there are two main mobility transitions associated with the glass transition and the melting. In this case, to determine the mobility transition temperatures, the *I*_DF‑MSE_^N^ vs *T* curves can be fitter by a phenomenological sigmoidal Boltzmann function such as^34,62^

2 $$I_{DF-MSE}^{N}\left(T\right)=f_{A}\frac{1}{1+e^{-\left(T-T_{A}\right)/\sigma_{T_{A}}}}+f_{B}\frac{1}{1+e^{-\left(T-T_{B}\right)/\sigma_{T_{B}}}}$$

where *T*_A(B)_ is the transition temperature and σ_TA(B)_ is proportional to the slope of the *I*_DF‑MSE_^N^ vs temperature (*T*) curve during the transition.

Figure 7C,D depicts *I*_DF‑MSE_^N^ vs *T* curves obtained for samples target, dry 0%, dry 3%, wet 0%, and wet 7% in the temperature range of 25 to 200 °C. A clear onset in the *I*_DF‑MSE_^N^ vs *T* intensity, rising from 0 to approximately 0.4, is observed in all samples. This behavior corresponds to the increase in segmental mobility associated with the glass transition of the samples. Additionally, a slight increase in the slope of the *I*_DF‑MSE_^N^ vs *T* curves above 175 °C is noted, which is likely due to enhanced mobility of segments located in the amorphous–crystalline interphase, since no sharp melting transition was detected within the studied temperature range (note that 200 °C is the equipment’s upper temperature limit). Therefore, eq 2, with two components, was used to fit the curves; however, only the parameters related to transition A (corresponding to the glass transition) were reliable and are presented in Table 5.

**Table 5:** Parameters Extracted from Fitting the *I*_DF‑MSE_^N^ vs *T* Curves of Different Samples Using Eq 2 (Columns 2 and 3), Figure 5C,D, and Eq 3, Figure 5E,F

sample	*f*_A_	*T*_A_ (°C)	σ_T_A__	τ_0_ (s)	⟨*E*_A_⟩ (kJ/mol)	σ_EA_ (kJ/mol)
target	0.36 ± 0.02	108 ± 1	14.6 ± 0.9			
dry 0%	0.27 ± 0.03	65 ± 1	17.9 ± 1.6	(1.7 ± 0.5) × 10^–12^	40 ± 4	4 ± 0.5
dry 3%	0.28 ± 0.01	99 ± 1	13.2 ± 0.3	(3.0 ± 0.5) × 10^–14^	54 ± 6	6 ± 0.5
wet 0%	0.36 ± 0.01	82 ± 1	16.2 ± 0.3	(8.0 ± 0.5) × 10^–14^	47 ± 7	5 ± 0.5
wet 7%	0.25 ± 0.01	117 ± 1	11.7 ± 0.6	(1.7 ± 0.5) × 10^–21^	110 ± 20	10 ± 0.5

Remarkably, the transition temperatures obtained for the samples are clearly distinguishable, showing an increase in the CDI content for both the dry and wet routes. Furthermore, for the wet 7% sample, the transition temperature becomes higher than that of the target sample. Considering that chain extension can plausibly lead to variations in the transition temperature, this result provides initial evidence that the reaction with CDI is associated with an increase in the chain length. However, it is important to emphasize that the *I*_DF‑MSE_^N^ vs *T* curves are also dependent on the filter time used, with longer filter times leading to higher observed transition temperatures and affecting the transition width σ_T_.

Thus, to discuss the dynamic constraints in the polymer chain induced by CDI, it is necessary to further explore the DF-MSE experiment to extract intrinsic motion parameters. This can be achieved based on ref 34, where an analytical function for the *I*_DF‑MSE_^N^ vs *T* curve was derived using the so-called Anderson and Wiess theory. Following these lines, the *I*_DF‑MSE_^N^ can be obtained as a function of the strength of the ^1^H–^1^H dipolar interaction, represented by its second moment *M*_2_, the correlation time of the motion τ_C_, and the filter time *t*_f_ used in the DF-MSE experiment as

3 $$I_{DF-MSE}^{N}\left(t_{f},\tau_{c}\right)=\exp\left(-M_{2}\tau_{c}^{2}\left(\exp\left(-\frac{t_{f}}{\tau_{c}}\right)+\frac{t_{f}}{\tau_{c}}-1\right)\right)$$

To model the temperature dependence of the *I*_DF‑MSE_^N^ intensity, it is assumed that the segmental mobility is governed by a thermally activated process, allowing the experimental behavior to be described in terms of physicochemical parameters associated with molecular dynamics.

Thus, while *t*_f_ is an experimental parameter, *M*_2_ can be obtained as the second moment of Gaussian decay of the signal at low temperatures. The correlation time τ_c_ can be related to the temperature assuming a given activation function τ_c_ = *f*(*T*). For instance, considering an Arrhenius-type activation behavior, the correlation time is expressed as $\tau_{c}=\tau_{0}\exp\left(\frac{E_{A}}{RT}\right)$ , where *R* is the ideal gases constant, *E*_A_ is the activation energy of the motion, and τ_0_ is the phonon correlation time.

Within this assumption, the *I*_DF‑MSE_^N^(*t*_f_, τ_c_) function can be rewritten as *I*_DF‑MSE_^N^(*t*_f_, *T*), parametrized by the activation parameters *E*_A_ and τ_0_, which can then be used to fit the experimental *I*_DF‑MSE_^N^ vs *T* curves. Moreover, since a single activation energy is not expected in glassy systems, a normal distribution of activation energies centered at ⟨*E*_A_⟩ with rms width σ_E_A__ can be incorporated, allowing for a three-parameter fitting procedure. Additionally, because *t*_f_ is an experimental parameter, the *I*_DF‑MSE_^N^ vs *T* curves can be acquired at different filter times, enabling a multicurve fitting approach to improve the reliability of the fitting results.

Figure 7E shows a set of *I*_DF‑MSE_^N^ vs *T* data acquired with filter times *t*_f_ of 50 μs, 100 μs, and 200 μs for the sample dry 0%. The solid lines represent the best-fit curves using eq 3, employing the same τ_0_, ⟨*E*_A_⟩, and σ_E_A__ parameters. The same procedure was applied to the samples dry 3%, wet 0%, and wet 7%, the results being shown in Figure S4 of the Supporting Information and the obtained fitting parameters listed in Table 5. Figure 7F shows a graphical representation of the normal activation energy distributions obtained from these fittings.

As can be observed in both Table 5 and Figure 7F, there is a clear trend of increasing ⟨*E*_A_⟩ and σ_E_A__ for the samples with higher CDI content, for both dry and wet routes. While the increase in ⟨*E*_A_⟩ can be associated with the increase in the overall chain stiffness, the increase in σ_E_A__ states for an increase in the degree of dynamic heterogeneity during the glass transition (*T*_g_). Both results indicate a molecular alteration with the increase of CDI amounts, as an outcome of the reaction between PA6 chains and CDI. Possibly, this increase in *T*_g_ is associated with the linear coupling mechanism and with an increase in the polydispersity index, whereas ramifications tend to increase the free volume and, consequently, increase the chain mobility.

Another way to verify if the CDI and PA6 reaction occurs through a linear chain extension or branching formation is by monitoring the formation of tertiary carbons since the PA6 monomer only has secondary carbons. The formation of branches would lead to the formation of tertiary carbons, which can be detected by ^13^C NMR analyses (Figure 8).

**Figure 8:**
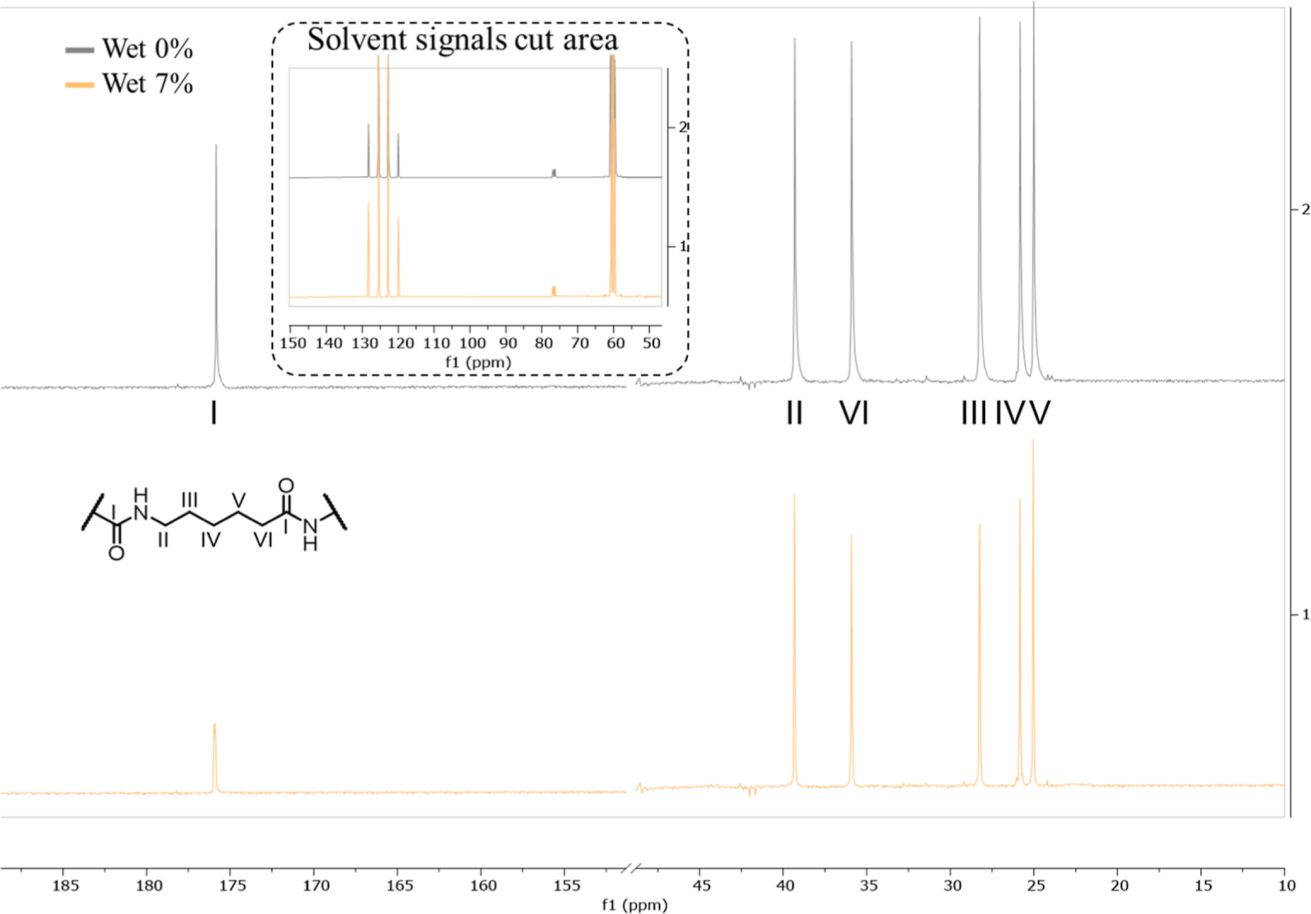
^13^C NMR spectrum in TFA/CDCl_3_ of the wet 0% samples (top) and wet 7% samples (bottom). Peak assignments are based on literature data ref 64.

The spectra presented in Figure 8 demonstrate that there are no changes in the carbon chemical shifts that could justify the formation of N-branching. This result is consistent with the extensive literature on the use of CDIs as coupling agents for amino acids, in which no parallel reactions leading to cross-linking or branching have been reported.^65–67^ Therefore, the data obtained suggest that the predominant chain extension mechanism of PA6 with CDI is linear, and a reaction mechanism between CDI and PA6 is proposed, as shown in Figure 9.

**Figure 9:**
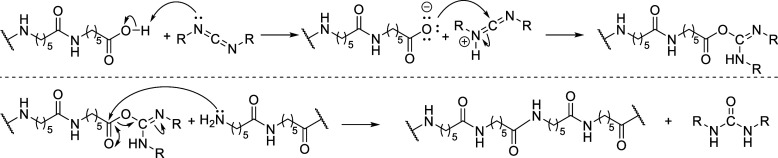
Proposed mechanism for the coupling of the acid terminus with the amine terminus mediated by CDI.

According to Schotman,^68^ carboxylic acids can react with CDIs in coupling reactions. In the case of polyamides, the carboxylic acid terminus is first activated, converting it into a good leaving group, which is subsequently attacked by the amine terminus, thereby coupling the two distinct chain ends of PA6 and resulting in a polyamide with the combined molar mass of the two original segments (Figure 9). The proposed mechanism supports the results obtained, which indicate chain extension, especially with the coupling of lower molar mass polymers.

In order to corroborate the proposed chain extension mechanism, DFT calculations were performed by using simplified reactants to mimic the reactive sites of the polymer, aiming to evaluate the feasibility of the transition state energy barriers (Figure 10). In addition to the terminal chain extension pathway, an alternative route involving the possibility of N-branching was also investigated (Figure 10). Regarding the terminal coupling pathway, the initial energy barrier corresponds to a “concerted transition state” (TS1) involving the simultaneous proton transfer from the carboxylic acid and nucleophilic attack on the CDI carbon. This step requires an activation free energy of 23.0 kcal·mol^–1^, leading to intermediate C. The latter undergoes nucleophilic attack by the amine through the transition state TS2 (23.5 kcal·mol^–1^), yielding intermediate E. A subsequent proton transfer and conformational reorganization afford intermediate F. This intermediate then proceeds through a low-barrier concerted extrusion of urea H via transition state TS3 (2.5 kcal·mol^–1^), furnishing amide product G with an overall reaction free energy of −27.3 kcal·mol^–1^. In contrast, a potential competing pathway leading to N-branching initiated by the proton transfer of intermediate G on C (in place of amine B), resulting in intermediate X1, was found to be highly endergonic, with a free energy change of 87.2 kcal·mol^–1^, well above the barrier for TS1. This indicates that the N-branching pathway is energetically unfeasible under the conditions studied. Taken together, the computed energy barriers support a highly favorable linear chain growth pathway, consistent with the proposed mechanism.

**Figure 10:**
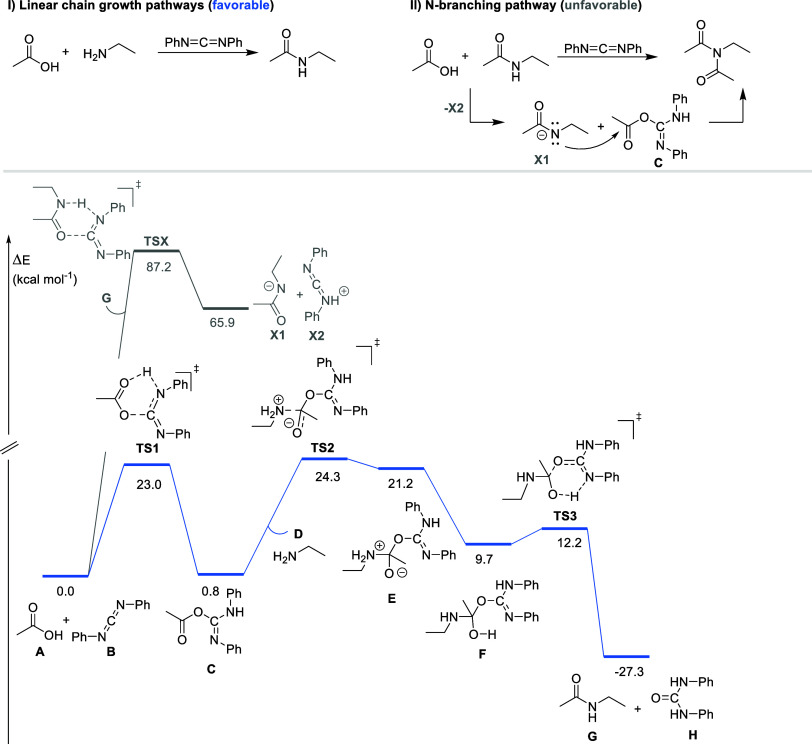
Computed energy profile for the chain extension reaction mediated by CDI. Free energies (in kcal·mol^–1^) computed at the M06-2X/def2-TZVP (SMD, ε = 3.2) level.

The better performance of CDI in a moist environment may be related to a competition between water and CDI to react with PA6, reducing the amount of hydrolysis of PA6, with CDI reacting with water to form urea, as shown in Figure 11. This, in turn, decreases the number of hydrolysis reactions, or alternatively, water assists in the deprotonation of the carboxylic acid and stabilizing the generated charges, thus facilitating chain extension.

**Figure 11:**
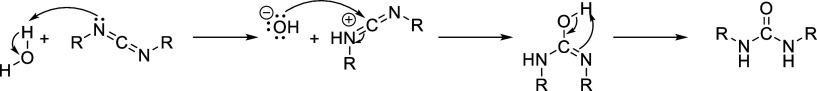
Reaction mechanism between CDI and water.

### 3.5 CDI Effect on the Mechanical Properties

One of the main characteristics of fibers is their mechanical properties. Therefore, Young’s modulus, elongation at break, and yield stress are presented in Figure 12 and Table 6.

**Figure 12:**
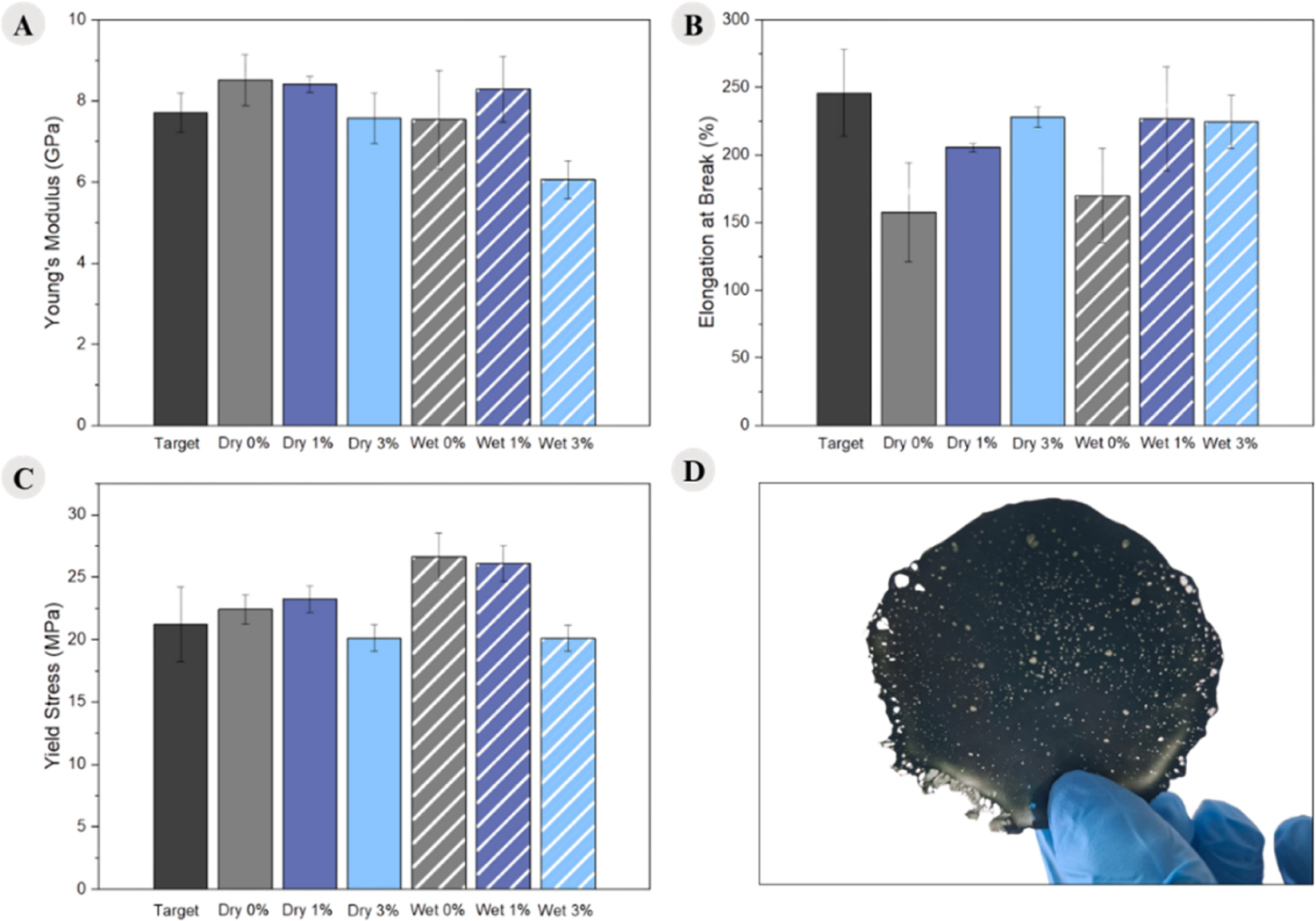
Mechanical tests: (A) Young’s modulus; (B) elongation at break; (C) yield stress; (D) wet 7% film.

**Table 6:** Mechanical Test Data (Young’s Modulus, Elongation at Break, and Yield Stress)

	Young’s modulus (GPa)	elongation at break (%)	yield stress (MPa)
target	7.71 ± 0.49	245.81 ± 31.87	21.23 ± 2.98
dry 0%	8.51 ± 0.63	157.77 ± 36.58	22.42 ± 1.18
dry 1%	8.41 ± 0,20	205.66 ± 3.20	23.25 ± 1.08
dry 3%	7.58 ± 0.62	227.90 ± 7.66	20.13 ± 1.08
wet 0%	7.54 ± 1.22	169.87 ± 34.98	26.66 ± 1.90
wet 1%	8.29 ± 0.81	226.87 ± 38.45	26.10 ± 1.42
wet 3%	6.06 ± 0.46	224.55 ± 19.48	20.9 1.05

Based on the data presented in Table 6 and Figure 12A,B, it can be observed that, for both types of samples, there is a tendency for decreasing Young’s modulus as well as yield stress with increasing CDI content. When these results are compared with the XRD patterns shown in Figure 4A,B, one possible explanation is a crystallographic phase transition from the α to the γ phase induced by the recycling process and the incorporation of CDI. These crystalline phases are directly associated with the mechanical properties discussed. The γ phase of PA6 exhibits lower Young’s modulus and lower yield stress compared to the α phase.^43^ Another possible explanation is associated with molar mass broadening, once the smaller chains increase free volume by spacing out the polymer chains, resulting in a higher ductility.^69^ This effect was also reported by Rosenbloom et al.^69,70^ They synthesized a polystyrene–polyisoprene block copolymer with controlled molar mass and molar mass distribution to investigate their influence on the polymer’s properties. The results showed that samples with a broader molar mass distribution exhibited less stiff behavior, characterized by a lower Young’s modulus and a lower yield stress compared to samples with similar average molar mass.

In addition, Table 6 and Figure 12C show a trend toward increasing elongation at break with an increasing CDI content. For the wet samples, the strain stress increased from 169% to 0% CDI to 224% to 3% CDI, while for the dry samples, it increased from 157% to 227% to 3% CDI, indicating that the material has become more ductile. Moreover, both types of samples nearly reached the elongation at break of the target material at 3% CDI. This behavior may be associated with the crystalline phases of PA6, since the γ phase exhibits more ductile behavior than the α phase,^43^ or it may also be related to the increase in molar mass caused by the chain extension, because longer chains cause an increase in the number of entanglements, which results in a higher elongation.^71^ A similar observation was reported by Promnil et al.^71^ They electrospun PLA with two different molar masses and observed that fibers produced from the higher-molar-mass PLA exhibited greater elongation at break, attributed to a higher number of chain entanglements.

As can be observed, mechanical tests were not performed for samples containing 5% and 7% CDI. This was due to the inability to produce suitable, “hole-free films” for testing, as shown in Figure 12D. This may be attributed to the higher content of reaction byproducts (a urea-like compound with a melting temperature of approximately 130 °C–190 °C) present in samples with higher CDI contents. This compound has a boiling point lower than the pressing temperature of PA6 (240 °C). Thus, during the hot-pressing process, the byproduct may evaporate and, through diffusion processes, escape from the PA6 matrix, resulting in hole formation. However, since this material would be reprocessed for fiber formation, the presence of the byproduct may not significantly affect the final fiber properties.

## 4 Conclusion

The rheology analyses indicated that for both dry and wet samples, there was an increase in complex viscosity, suggesting an increase in molar mass. It is worth noting that the dry sample with 3% CDI showed the best performance among the dry samples, achieving a 40% viscosity increase compared to the one without CDI. Among the wet samples, the 7% CDI sample exhibited the best overall performance, with a 75% increase compared to the 0% CDI sample, surpassing the target material’s viscosity. This indicates that CDI is more effective in water-containing environments.

It was also observed that the shear-thinning behavior of PA6 intensified with increasing CDI content, being more pronounced in the dry samples. This suggests a possible broadening of the molar mass distribution or the formation of more branches at higher CDI concentrations. However, the increase in *T*_g_ with CDI addition indicates a reduction in the free volume, which is associated with a linear increase in molar mass without the presence of branching. Furthermore, ^13^C NMR analyses indicate no formation of tertiary carbon, corroborating the absence of branching, and DFT calculations corroborate with both analyses showing that linear chain extension has minor energy barriers compared to the branching extension. Based on this, a reaction mechanism between PA6 and CDI that leads to the linear chain extension of PA6 was proposed, with water helping in the extension process. Flow-induced crystallization results show that the addition of CDI reduces chain mobility, hindering nucleation and crystallization kinetics, supporting the results indicating an increase in the molar mass. Mechanical tests show that the addition of CDI improved the strain stress, and the interesting thing is that the dry sample improved about 70%, and the wet samples improved 55%, even though the best condition according to the rheological data of the wet route (7%) was not tested.

## Supplementary Material



## Data Availability

All data supporting the findings of this study are available within the manuscript and its Supporting Information files.

## Notes

Amplitude sweep curves for samples processed via dry and wet routes, carbon 13 NMR spectrum of CDI, and temperature-dependent DF MSE intensity analysis with activation energy distributions (PDF)
