# Supplementary material for: Circularity in Polyamide Textiles: Enhancing Recycled Polymer Molar Mass with Carbodiimide Linear Coupling
Source: ACS Omega. 2026 Mar 12;11(11):18189–205. doi: 10.1021/acsomega.5c13446 (PMC13019264; doi:10.1021/acsomega.5c13446)
Supplement: Supplementary file 1 [file ao5c13446_si_001.pdf]

Circularity in Polyamide Textiles: Enhancing Recycled Polymer Molar Mass with Carbodiimide Linear Coupling

Graziela S Baccarin[1], Mateus O Costa[1], Rodrigo H dos S Garcia[3], Bruno Trebbi[3], Eduardo R de Azevedo[3], Marco A B Ferreira[1], Lucas H Staffa[2], Sandra A Cruz[1]*

[1] Federal University of São Carlos (UFSCar), Department of Chemistry (DQ), Rodovia Washington Luis, km 235, 13565-905, São Carlos, SP, Brazil

[2] Federal University of São Carlos (UFSCar), Department of Materials Engineering (DEMA), Rodovia Washington Luis, km 235, 13565-905, São Carlos, SP, Brazil

[3] University of São Paulo (USP), Institute of Physics of São Carlos (IFSC) , Avenida Trabalhador São Carlense, 400, 13566-590, São Carlos, SP, Brazil

*Corresponding author, Tel.: +55 16 3351-8080; E-mail address: sandra.cruz@ufscar.br (S.A. Cruz).

Supplementary Information

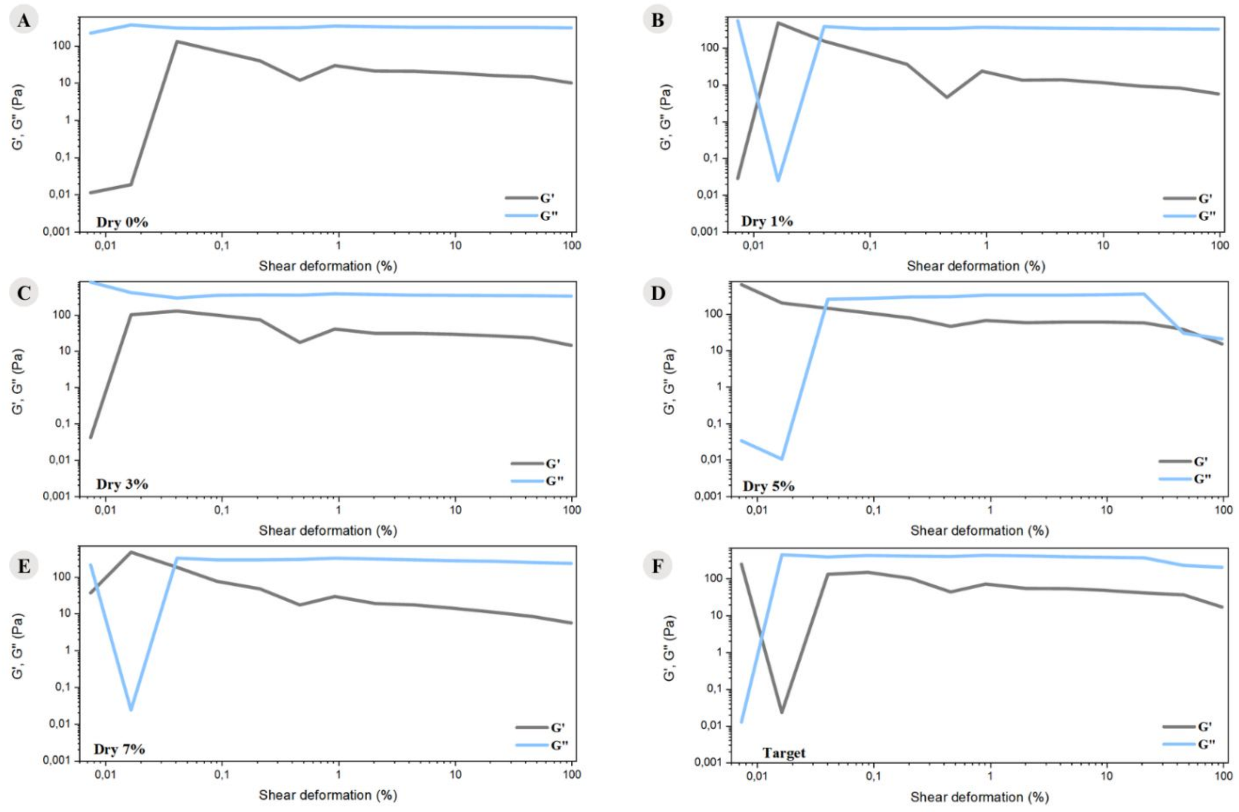

Figure S1 Amplitude sweep curves for the samples: A- Dry 0%, B- Dry 1%, C- Dry 3%, D- Dry 5%, E- Dry 7%, and F- Target.

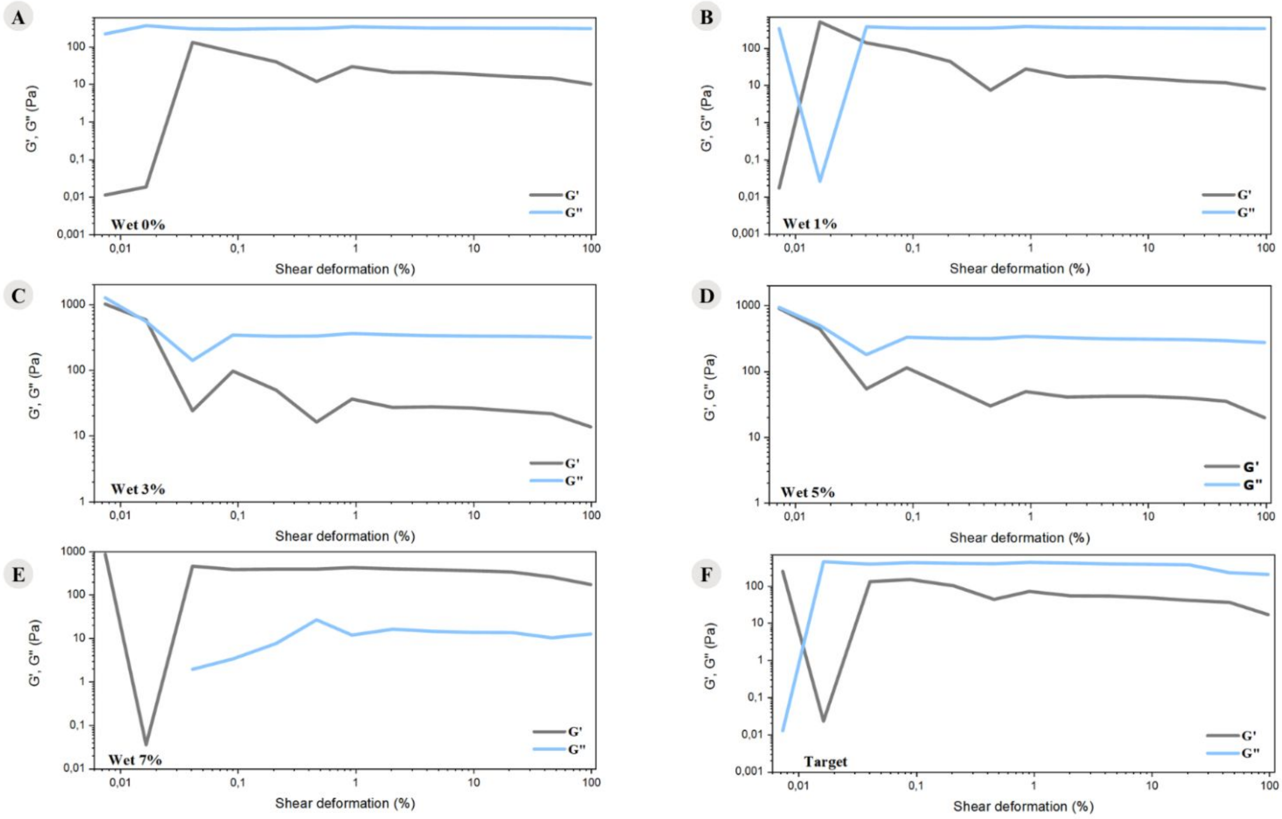

Figure S2 Amplitude sweep curves for the samples: A- Wet 0%, B- Wet 1%, C- Wet 3%, D- Wet 5%, E- Wet 7%, and F- Target.

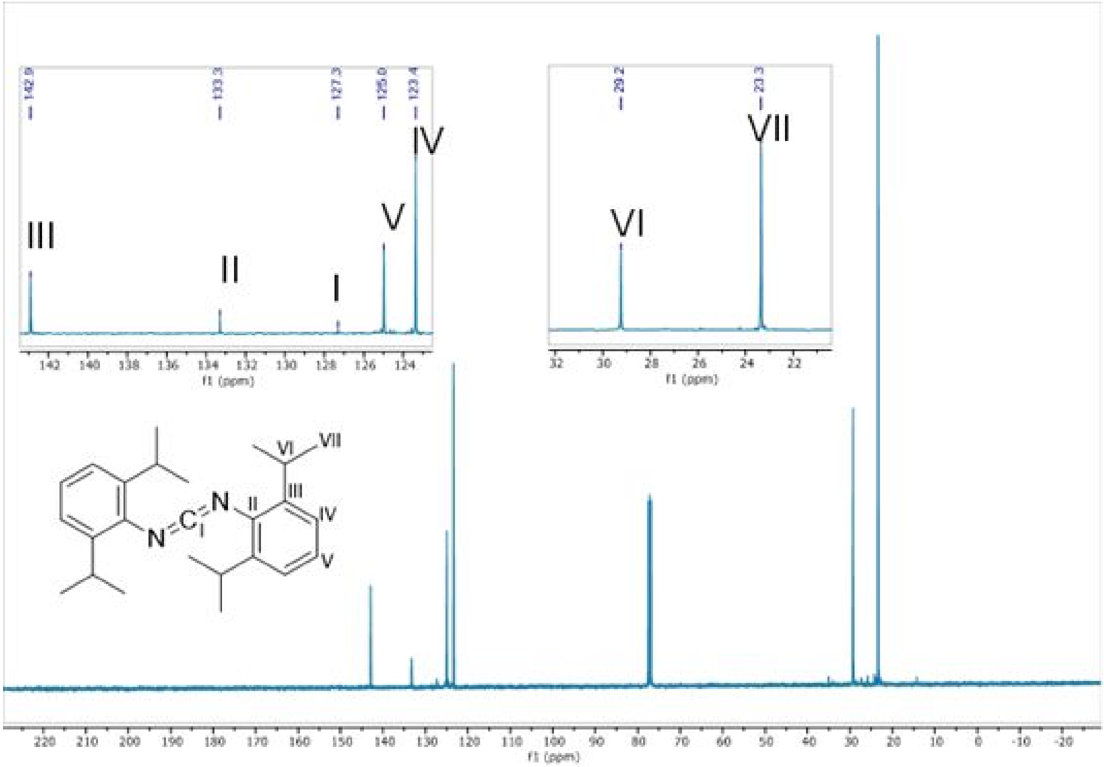

Figure S3 ^{13}C NMR spectrum of CDI in $CDCl_3$ (100 MHz).

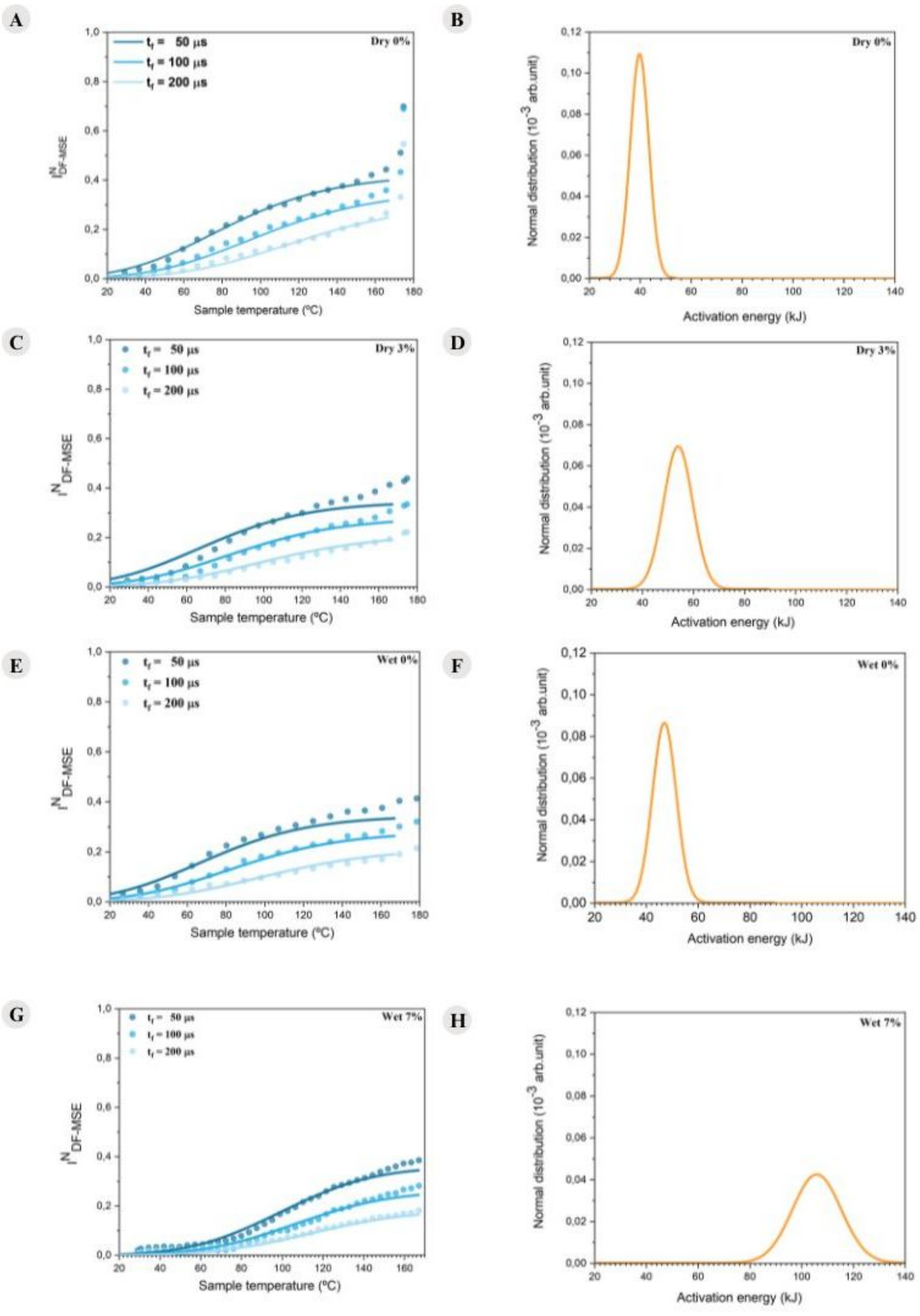

Figure S4 Normalized DF-MSE intensities as a function of temperature for different filter time (t_f) for sample (A) Dry – 0%, (C) Dry – 3 %, (E) Wet – 0%, (G) Wet – 7 % and normal distribution as a function of activation energy for sample (B) Dry – 0%, (D) Dry – 3 %, (F) Wet – 0% and (H) Wet – 7 %.
